# The Impact of Vision Impairment on Self-Reported Falls Among Older US Adults: Cross-Sectional and Longitudinal Study

**DOI:** 10.2196/68771

**Published:** 2025-07-31

**Authors:** Kasem Seresirikachorn, Rachasak Somyanonthanakul, Matthew Johnson, Panisa Singhanetr, Jiraporn Gatedee, David Friedman, Nazlee Zebardast

**Affiliations:** 1 Massachusetts Eye and Ear Infirmary, Harvard Medical School, 243 Charles Street, Boston, MA, 02114, United States, 1 617-573-3202; 2 Department of Ophthalmology, College of Medicine, Rangsit University, Rajavithi Hospital, Bangkok, Thailand; 3 Data Science and Innovation, College of Interdisciplinary Studies, Thammasat University, Pathum Thani, Thailand; 4 Mettapracharak Eye Institute, Mettapracharak (Wat Rai Khing) Hospital, Nakhon Pathom, Thailand; 5 Faculty of Medical Technology, Rangsit University, Pathum Thani, Thailand

**Keywords:** elderly, falls, glaucoma, Health and Retirement Study, vision impairment

## Abstract

**Background:**

Falls are the leading cause of injury among older adults, with vision impairment recognized as a significant risk factor. However, many existing studies have been limited by small sample sizes, retrospective designs, or insufficient adjustment for confounding factors. To overcome these limitations, we used data from the University of Michigan’s Health and Retirement Study (HRS) to analyze the association between self-reported vision and fall risk among older adults in a large, nationally representative sample.

**Objective:**

The objective of this study was to investigate the association between vision impairment and falls and assess whether subjective vision impairment predicts future falls in older adults.

**Methods:**

This cross-sectional and longitudinal analysis used data from the HRS (1996‐2020) to assess the relationship between self-reported vision, glaucoma history, and falls among US adults aged 65 years and older. HRS uses a biennial, multistage area probability sample survey design, collecting data with community-dwelling individuals followed up every 2 years until death, tracking health, economic, and social outcomes. Multivariate logistic regression was used to analyze associations between self-reported vision and self-reported falls in the past 2 years.

**Results:**

A total of 38,835 respondents contributed 117,834 observations. The weighted proportion of participants reporting falls was 37.9% (95% CI 37.7%‐40.1%). Significant risk factors for falls included overall eyesight impairment (adjusted odds ratio [aOR] 1.36, 95% CI 1.20‐1.56), distance vision impairment (aOR 1.37, 95% CI 1.32‐1.42), near vision impairment (aOR 1.33, 95% CI 1.27‐1.37), and glaucoma (aOR 1.15, 95% CI 1.07‐1.24). A similar association was observed for serious falls, where overall eyesight impairment (aOR 1.20, 95% CI 1.03‐1.44), distance vision impairment (aOR 1.14, 95% CI 1.07‐1.22), near vision impairment (aOR 1.12, 95% CI 1.05‐1.18), and glaucoma (aOR 1.15, 95% CI 1.05‐1.26) were significant. In longitudinal analyses, overall vision impairment (aOR 1.23, 95% CI 1.16‐1.29), distance vision impairment (aOR 1.27, 95% CI 1.20‐1.38), near vision impairment (aOR 1.23, 95% CI 1.19‐1.32), and glaucoma (aOR 1.25, 95% CI 1.13‐1.37) increased the risk of future falls. Reported overall vision was significantly associated with the number of falls in both the same (*P*<.001) and subsequent (*P*<.001) survey cycles.

**Conclusions:**

Both distance and near vision impairment, as well as glaucoma, are associated with a higher risk of falls in older adults and present possible areas for intervention and prevention.

## Introduction

Falls are the leading cause of injury among adults ages 65 years or older [1]. Nearly 30% of older adults fall each year, and 10% report injury from a fall [2]. In the United States in 2018, there were 36 million reported falls, resulting in 3 million emergency department visits, nearly 1 million hospitalizations, and 32,000 deaths [3]. Approximately 1 in 10 falls in older adults results in serious injury, such as hip fracture or traumatic brain injury, and the annual cost of falls in the United States is more than US $50 billion [4-6]. Globally, falls are the 13th leading cause of death, and the prevalence of falls is increasing as the population gets older [7]. Fortunately, multifactorial intervention strategies such as medical staff and patient education, providing supplies for hip protection, and sensory optimization by providing hearing aids have all been able to successfully reduce the risk of falling [5,7,8]. Home modifications to improve safety, such as changing floor coverings, removing loose carpets, fitting handrails, maintaining steps and ramps, and improving lighting, can reduce falling hazards in homes.

Falls are significant, life-altering health events with modifiable risk factors such as impaired balance, polypharmacy, and poor vision [9-11]. Impaired vision has been shown to be a risk factor for falls in older adults. In 1998, the Beaver Dam Eye Study showed that impaired visual acuity was associated with an increased risk of falling, and older adults in the worst visual acuity group were significantly more likely to have fallen 2 or more times in the past year [12]. The Blue Mountains Eye Study similarly found a significant relationship between low visual acuity and falls. In addition, they reported that decreased contrast sensitivity and reduced visual field (VF) increased the risk of falls and proposed that the presence of a cataract may explain this association [13]. In the Salisbury Eye Evaluation study, only reduced peripheral VF was associated with the risk of falling, while visual acuity, contrast sensitivity, and stereo acuity were not after adjusting for potential confounders [14]. Other studies support a relationship between impaired vision and falls but differ as to which components of visual function increase fall risk [5,9,15-17].

These studies provided valuable information that broadly supports impaired vision as a modifiable risk factor for falls; however, many of these studies had limitations due to small sample sizes, retrospective analyses, or the inability to adjust for confounders. Even the most extensive studies to date have data spanning only 1‐2 years and are generally limited to local communities or geographic regions [12-14].

To address these limitations, we used the University of Michigan Health and Retirement Study (HRS) data. The HRS is a diverse, nationally representative longitudinal panel study of US adults ages 50 years and older, followed every 2 years for over 20 years [18]. Data on fall incidence and self-reported vision impairment were collected from over 25,000 respondents over a period of two decades. [7]. Self-reported vision has been established as a practical proxy measure for visual function, demonstrating a strong relationship with various objective visual function assessments [19,20]. This indicates that self-reported vision can serve as a reliable indicator of visual acuity. Furthermore, self-reported vision assessments offer a valuable advantage in under-resourced areas, where accessible and convenient tools for vision evaluation are scarce. By leveraging self-reported vision data, researchers and health care professionals can overcome logistical barriers and better understand vision-related needs in these communities. A better understanding of what aspects of vision impairment increase fall risk will help clarify promising vision interventions to decrease the rate of falls among older adults.

The objective of this study was to investigate the relationship between various aspects of self-reported vision impairment and falls and prospectively assess whether subjective vision impairment is predictive of future falls in a large and diverse longitudinal population-based study of older adults.

## Methods

### Data Collection

Data were from the HRS database, a longitudinal panel study that surveys a nationally representative sample of Americans aged 50 years and older every two years, with follow-up continuing until death. The HRS uses a biennial, multistage area probability sample survey design, using a combination of in-person and telephone interviews to collect data. The survey focuses on community-dwelling individuals. This longitudinal design enables the tracking of participants’ health, economic, and social outcomes over time, providing a comprehensive understanding of aging and retirement in America. The HRS is supported by the National Institute on Aging (NIA U01AG009740) and the Social Security Administration. For this study, we used data from 14 waves of the HRS (1996‐2020).

The analytic subsample for this study comprises community-dwelling respondents aged 65 years and older from the 1996‐2020 HRS survey rounds, as the falls questionnaire was only administered to participants at or older than this age threshold, with all included variables having item nonresponse rates below 10%.

### Ethical Considerations

Since the HRS database is deidentified and publicly accessible, this study was exempt from the Mass General Brigham institutional review board approval and patient permission was waived. The study adhered to the tenets of the Declaration of Helsinki.

### Primary Outcomes and Definitions of Variables

The primary outcome measures included self-reported falls, serious injury falls, and the number of falls. Participants were asked if they had fallen in the past two years. If they answered “yes,” they were then asked how many times they had fallen and whether any of the falls were serious. Serious injury falls were defined as those requiring medical treatment.

Ophthalmic parameters were acquired from the HRS survey assessments and included overall eyesight, distance vision, and near vision (see Multimedia Appendix 1), each rated on a scale from 1 to 5, with scores of 4 (fair) and 5 (poor) considered indicative of impaired vision. For overall eyesight, a score of 6 could be assigned for patients with legal blindness. The history of glaucoma was based on whether participants reported ever being treated for glaucoma.

The HRS also collected information on age, gender, race, level of education, marital status, self-reported diagnoses of hypertension, diabetes, stroke, heart conditions (including heart attack, coronary heart disease, angina, congestive heart failure, or other heart problems), arthritis or rheumatism, smoking and alcohol consumption status, psychiatric medication use, exercise activities, and household income.

### Study Timepoints of Data Analysis

The data were analyzed cross-sectionally and longitudinally to confirm the correlations between ophthalmic parameters and falls. Each participant observation was analyzed as individual encounters in both analyses. In the cross-sectional analysis, self-reported ophthalmic parameters and falls were ascertained from the same 2-year interview cycle. Each completed survey was counted as 1 entry. In the longitudinal analysis, the self-reported ophthalmic parameters were obtained during the survey and analyzed with the reported falls recorded in the patient’s survey 2 years later.

### Statistical Analysis

Data are presented as base-year weighted proportions, accounting for the Health and Retirement Study adjustments for attrition and sampling error. Descriptive statistics were used to summarize participant data on falls outcomes, including falls, no falls, serious falls, and nonserious falls. We used *t* tests for continuous variables and *χ*^2^ tests for categorical variables to evaluate differences between outcomes. We used univariable and multivariable logistic regression models to determine the cross-sectional and longitudinal association between ophthalmic parameters and falls, adjusting for age and other potential confounders by calculating the odds ratio (OR) and their corresponding 95% CI. For the cross-sectional model, we assessed the association between vision status and reported falls in the same 2-year survey cycle, while for the longitudinal model, we considered falls reported in the subsequent 2-year survey cycle.

All variables with a *P* value of <.05 in the univariable analyses (including age, sex, Hispanic ethnicity, race, education, marital status, hypertension, diabetes, heart disease, stroke, arthritis, smoking, alcohol status, psychiatric medications, degree of exercise, and household income) were included in multivariable logistic regression models. Separate multivariable regression models were used to determine the relationship between each ophthalmic parameter and falls and serious falls. In addition, the association between the degree of vision impairment and the number of falls was assessed using a 1-way analysis of variance. All models were analyzed using generalized estimating equations for multiple observations from the same participant and multiple imputation techniques to replace the missing data. Data analysis was done with IBM SPSS Statistics 22.0 for Microsoft Windows and *P* values <.05 were considered statistically significant.

## Results

### Demographics of Study Sample

A total of 38,835 respondents contributed to 117,834 participant-observations (see Tables1  2). The total number of observations in which participants reported a total of 40,477 falls and 13,471 serious falls. The weighted proportion of participants who reported any falls was OR 37.9% (95% CI 37.7%‐40.1%), while the proportion reporting serious falls was OR 30.9% (95% CI 30.7%‐31.1%). Furthermore, the weighted proportion of participants who experienced falls in the future was OR 36.5% (95% CI 35.8%‐37.2%), and OR 32.5% (95% CI 32.2%‐32.9%) for future serious falls. Falls increased with older age, and over half of those aged 90 years and older reported falling. The proportion of falls qualifying as serious also increased with age. White people had the highest fall rate, and a higher proportion of falls and serious falls was observed in females and individuals with lower levels of education. In total, 50% of patients with a history of stroke reported falling, with one-third of the falls being serious falls.

**Table 1.** Baseline characteristics of study participants comparing those who reported falls versus no falls (total number of participant-observations).

Characteristic	Participants with any self-reported falls (n=40,477)	Participants with no self-reported falls (n=77,357)	*P* value
Total (%), OR^a^ (95% CI)	37.9 (37.7‐40.1)	62.1 (61.9‐62.3)	—^b^
Age in years (%), OR (95% CI)			<.001
65‐69	28.5 (28.2‐28.7)	71.5 (71.2‐71.9)	
70‐74	30.9 (30.5‐31.4)	69.1 (68.6‐69.7)
75‐79	32.9 (32.3‐33.7)	67.1 (66.5-67.8)
80‐84	37.9 (37.5‐38.5)	62.1 (61.6-62.9)
85‐89	44.8 (44.2‐45.6)	55.2 (54.8-55.9)
90+	52.2 (51.6-52.7)	47.8 (46.8-48.4)
Sex (%), OR (95% CI)			<.001
Male	30.2 (29.8‐30.8)	69.8 (69.2‐70.3)	
Female	35.7 (35.3‐36.1)	64.3 (63.8‐64.8)
Ethnicity (%), OR (95% CI)			<.001
Hispanic	33.1 (31.8‐33.9)	66.9 (65.8‐67.9)	
Non-Hispanic	34.7 (34.1‐35.3)	65.3 (65.0‐65.8)
Race (%), OR (95% CI)			<.001
White	34.7 (34.1‐35.3)	65.3 (64.3‐66.4)	
Black or African American	28.9 (28.0‐29.9)	71.1 (70.1‐72.0)
Others^c^	30.7 (29.9‐31.8)	69.3 (68.6‐70.1)
Education (%), OR (95% CI)			<.001
<High school	35.1 (34.6‐35.7)	64.9 (63.8‐65.5)	
High school degree	33.2 (32.8‐33.5)	66.8 (65.8‐67.2)
>High school degree	31.7 (31.1‐32.3)	68.3 (67.8‐68.8)
Marital status (%), OR (95% CI)			<.001
Married	61.0 (60.8-61.2)	39.0 (38.9-39.1)	
Widowed, separated, or divorced	59.3 (59.0-59.8)	40.7 (40.2-41.3)
Never married	38.7 (38.2-39.2)	61.3 (60.9-61.7)
Comorbidities (%), OR (95% CI)			
Hypertension	35.7 (35.2-36.1)	64.3 (63.5-64.8)	<.001
Diabetes mellitus	39.7 (39.4-40.2)	60.3 (59.8-60.7)	<.001
Heart	42.0 (41.6-42.5)	58.0 (57.5-58.5)	<.001
Stroke	49.2 (48.3-50.0)	50.8 (50.3-51.5)	<.001
Arthritis (%), OR (95% CI)	36.8 (35.9‐38.0)	63.2 (61.9‐64.1)	<.001
Current smoking (%), OR (95% CI)	45.0 (44.3‐46.0)	56.0 (54.9‐57.1)	<.001
Active alcohol drinking (%), OR (95% CI)	55.2 (54.8‐55.7)	44.8 (44.0‐45.7)	<.001
Psychiatric medication (%), OR (95% CI)	62.1 (61.5‐62.8)	37.9 (37.1‐38.6)	<.001
Vigorous exercise (%), OR (95% CI)	70.6 (70.0‐71.2)	29.4 (28.8‐29.7)	<.001
Household income (US $), mean (95% CI)	64,319.1 (56,172.2‐71,816.3)	69,983.7 (45,562.1‐73,805.4)	<.001

a OR: odds ratio.

b Not applicable.

c Other races: Asian, Latino, and Native American.

**Table 2.** Baseline characteristics of study participants comparing those who reported serious falls versus no serious falls (total number of participant-observations).

Characteristics	Participants with any self-reported serious falls (n=13,471)	Participants with no self-reported serious falls (n=27,006)	*P* value
Total (%), OR^a^ (95% CI)	30.9 (30.7‐31.1)	69.1 (68.9‐69.3)	—^b^
Age in years (%), OR (95% CI)			<.001
65-69	21.4 (21.3‐21.5)	78.6 (78.3-78.8)	
70-74	31.2 (31.0-31.4)	68.8 (68.4-69.1)
75-79	40.6 (40.2-40.9)	59.4 (59.0-59.8)
80-84	42.6 (42.1-43.0)	57.4 (56.9-57.9)
85-89	43.9 (43.0-44.7)	56.1 (55.7-56.6)
90+	39.9 (39.3-40.6)	60.1 (59.1-61.1)
Sex (%), OR (95% CI)			<.001
Male	23.8 (23.7‐23.9)	76.2 (75.9‐76.5)	
Female	39.1 (38.9-39.4)	60.9 (60.3-61.5)
Ethnicity (%), OR (95% CI)			<.001
Hispanic	26.9 (26.3‐27.5)	73.1 (72.4‐73.9)	
Non-Hispanic	31.8 (31.2‐32.4)	68.2 (67.6‐68.8)
Race (%), OR (95% CI)			<.001
White	35.8 (35.2‐36.3)	64.2 (63.7‐64.8)	
Black or African American	20.0 (19.6‐20.5)	80.0 (79.7‐80.4)
Others^c^	19.9 (19.1‐20.8)	80.1 (79.6‐80.9)
Education (%), OR (95% CI)			<.001
<High school	31.9 (31.3‐32.7)	68.1 (67.5‐68.6)	
High school degree	34.1 (33.7‐34.5)	65.9 (65.5‐66.6)
>High school degree	26.9 (26.2‐27.4)	3.1 (72.7‐73.6)
Marital status (%), OR (95% CI)			<.001
Married	31.8 (31.3‐32.3)	68.2 (67.9‐68.6)	
Widowed, separated, or divorced	32.8 (32.3‐33.3)	67.2 (66.8‐67.9)
Never married	17.8 (17.3‐18.2)	82.2 (81.8‐82.6)
Comorbidities (%), OR (95% CI)			
Hypertension	32.8 (32.2-33.7)	67.2 (66.9-67.6)	<.001
Diabetes mellitus	32.3 (31.9-33.6)	67.7 (67.0-68.3)	<.001
Heart	35.0 (34.4-35.8)	65.0 (64.6-65.8)	<.001
Stroke	35.6 (34.5-36.3)	64.4 (64.1-64.7)	<.001
Arthritis (%), OR (95% CI)	33.3 (33.3‐34.3)	66.7 (65.9‐66.9)	<.001
Current smoking (%), OR (95% CI)	22.2 (21.6‐22.9)	77.8 (77.0‐78.7)	<.001
Active alcohol drinking (%), OR (95% CI)	28.6 (28.0‐29.8)	71.4 (70.8‐71.9)	<.001
Psychiatric medication (%), OR (95% CI)	35.1 (34.5‐35.7)	64.9 (64.1‐65.8)	<.001
Vigorous exercise (%), OR (95% CI)	40.2 (39.4‐40.9)	59.8 (59.1‐60.6)	<.001
Household income, mean (95% CI)	62,595.1 (53,582.5‐70,419.8)	64,289.8 (58,925.3‐69,643.1)	<.001

a OR: odds ratio.

b Not applicable.

c Other races: Asian, Latino, and Native American.

### Ophthalmic Parameters and Falls

Higher rates of any type of falls and serious falls were observed with worse eyesight (see Tables3  4). Approximately half of the participants who reported poor vision or blindness also reported falling, while 42.8% (95% CI 38.3%‐46.7%) of those with blindness had serious injuries associated with the fall. Of the participants with poor distant and near vision, 53.2% (95% CI 52.7%‐54.1%) and 50.2% (95% CI 49.9%‐50.6%) reported falls, respectively, and over a third of the falls were serious. In addition, falls were reported by 39.6% (95% CI 38.8%‐40.5%) of those who stated they had glaucoma.

**Table 3.** Ophthalmic parameters of the study sample between patients who reported falls and no falls (total number of participant-observations).

Characteristic	Participants with any self-reported falls (n=40,477), OR^a^ (%), (95% CI)	Participants with no self-reported falls (n=77,357), OR (%), (95% CI)	*P* value
Overall	37.9 (37.7‐40.1)	62.1 (61.9‐62.3)	—^b^
Overall eyesight			<.001
Excellent	28.7 (28.3-29.7)	71.3 (69.7-71.9)	
Very good	29.1 (28.8-29.5)	70.9 (70.1-71.8)
Good	32.2 (32.0-32.5)	67.8 (66.9-68.8)
Fair	38.9 (38.1-40.5)	61.1 (59.7-63.1)
Poor	51.1 (50.7-51.4)	48.9 (47.6-49.7)
Blind	48.3 (46.9-49.9)	51.7 (49.1-54.1)
Distance vision			<.001
Excellent	29.2 (28.1-30.0)	70.8 (69.4-71.9)	
Very good	29.8 (28.8-30.8)	70.2 (69.2-71.2)
Good	32.8 (31.8-33.8)	67.2 (66.2-67.9)
Fair	41.8 (41.0-42.9)	58.2 (56.8-59.3)
Poor	53.2 (52.7-54.1)	46.8 (44.9-47.8)
Near vision			<.001
Excellent	28.8 (28.3-30.0)	71.2 (69.8-71.9)	
Very good	29.8 (29.1-30.5)	70.2 (69.3-70.9)
Good	32.2 (32.1-33.0)	67.8 (66.7-68.4)
Fair	40.2 (39.7-41.0)	59.8 (58.5-60.8)
Poor	50.2 (49.9-50.6)	49.8 (47.9-51.1)
Glaucoma			<.001
Yes	39.6 (38.8‐40.5)	60.4 (58.5‐62.2)	
No	32.8 (32.0‐33.8)	67.2 (66.4‐69.0)

a OR: odds ratio.

b Not applicable.

**Table 4.** Ophthalmic parameters of the study sample between patients who reported serious falls and no serious falls (total number of participant-observations).

Characteristic	Participants with any self-reported serious falls (n=13,471), OR^a^ (%), (95% CI)	Participants with no self-reported serious falls (n=27,006), OR (%), (95% CI)	*P* value
Overall	30.9 (30.7‐31.1)	69.1 (68.9‐69.3)	—^b^
Overall eyesight			<.001
Excellent	31.7 (30.1‐33.7)	68.3 (65.3‐69.9)	
Very good	30.8 (29.6‐31.7)	69.2 (67.4‐70.2)
Good	31.7 (30.5‐33.0)	68.3 (67.0‐69.7)
Fair	33.3 (32.3‐34.8)	67.7 (65.1‐69.1)
Poor	37.9 (36.8‐39.6)	62.1 (60.4‐64.3)
Blind	42.8 (38.3‐46.7)	57.2 (51.3‐63.3)
Distance vision			<.001
Excellent	30.1 (29.7‐32.4)	69.9 (67.6‐71.4)	
Very good	31.7 (30.4‐33.3)	68.3 (66.7‐69.5)	
Good	31.8 (29.9‐32.7)	68.2 (67.3‐69.2)	
Fair	34.8 (33.5‐36.2)	65.2 (63.0‐66.6)	
Poor	39.1 (37.9‐41.2)	60.9 (58.4‐62.0)	
Near vision			<.001
Excellent	31.6 (30.5‐32.6)	68.4 (66.4‐69.2)	
Very good	31.2 (30.7‐32.9)	68.8 (67.1‐69.8)
Good	32.6 (31.4‐33.8)	67.4 (67.3‐68.9)
Fair	34.2 (33.5‐35.9)	65.8 (63.1‐67.6)
Poor	38.6 (37.6‐40.6)	61.4 (59.4‐63.1)
Glaucoma			<.001
Yes	37.2 (35.4‐39.5)	62.8 (60.3‐64.8)	
No	32.3 (32.1‐33.4)	67.7 (65.7‐68.9)

a OR: odds ratio.

b Not applicable.

### Cross-Sectional Analysis

**Figure 1.**
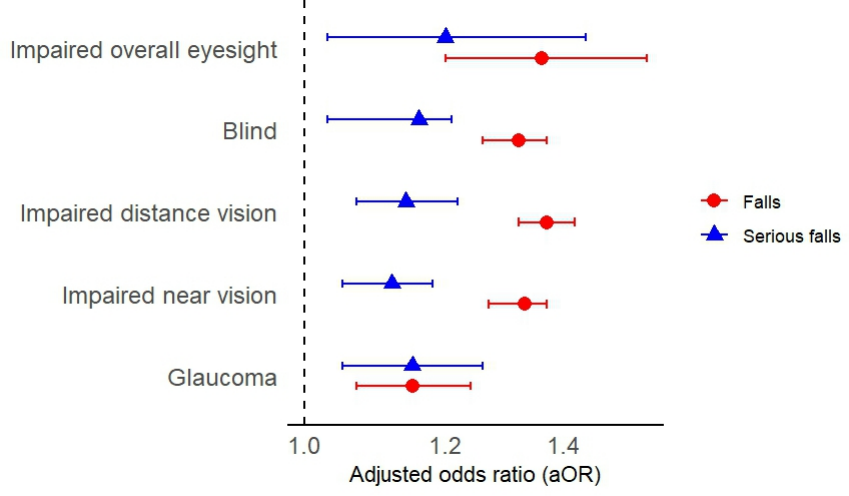
Forest plot illustrating the association between falls and ophthalmic parameters, based on a cross-sectional multivariable model using generalized estimating equations.

Cross-sectionally, vision impairment was associated with higher odds of falls: impaired overall eyesight (aOR 1.36, 95% CI 1.20‐1.56; *P*<.001), impaired distance vision (aOR 1.37, 95% CI 1.32‐1.42; *P*<.001), and impaired near vision (aOR 1.33, 95% CI 1.27‐1.37; *P*<.001) all conferred significantly increased odds of falls after adjusting for confounding factors (see Table 5 and Figure 1). The odds of serious falls increased in impaired overall eyesight with adjusted ORs of 1.20 (95% CI 1.03‐1.44; *P*<.001), while having overall eyesight qualifying as legal blindness increased the odds of falls (aOR 1.32, 95% CI 1.26‐1.37; *P*<.001) and serious falls (aOR 1.16, 95% CI 1.03‐1.21; *P*<.001) compared to not having impaired vision. Self-reported glaucoma increased the adjusted odds of falls (aOR 1.15, 95% CI 1.07‐1.24; *P*<.001) and serious falls (aOR 1.15, 95% CI 1.05‐1.26; *P*<.001).

**Table 5.** Cross-sectional model showing the association between falls and ophthalmic parameters.

Type of falls	OR^a^ (95% CI)	*P* value	Adjusted OR (95% CI)^b^	*P* value
Falls				
Overall eyesight				
Not impaired	Ref^c^	Ref	Ref	Ref
Impaired	1.60 (1.52‐1.73)	<.001	1.36 (1.20‐1.56)	<.001
Blind	2.03 (1.83‐2.44)	<.001	1.32 (1.26‐1.37)	<.001
Impaired distance vision	1.78 (1.70‐1.89)	<.001	1.37 (1.32‐1.42)	<.001
Impaired near vision	1.89 (1.69‐1.99)	<.001	1.33 (1.27‐1.37)	<.001
Glaucoma	1.33 (1.27‐1.41)	<.001	1.15 (1.07‐1.24)	<.001
Serious falls				
Overall eyesight				
Not impaired	Ref	Ref	Ref	Ref
Impaired	1.23 (1.11‐1.31)	<.001	1.20 (1.03‐1.44)	.045
Blind	1.49 (1.24‐1.89)	<.001	1.16 (1.03‐1.21)	.005
Impaired distance vision	1.22 (1.19‐1.27)	<.001	1.14 (1.07‐1.22)	.007
Impaired near vision	1.21 (1.14‐1.29)	<.001	1.12 (1.05‐1.18)	<.001
Glaucoma	1.24 (1.12‐1.33)	<.001	1.15 (1.05‐1.26)	.004

a OR: odds ratio.

b Adjusted for age, sex, Hispanic ethnicity, race, education, marital status, hypertension, diabetes, heart disease, stroke, arthritis, smoking, alcohol, activity, psychiatric medication, vigorous activities, and household income.

c Reference.

### Longitudinal Analysis

**Figure 2.**
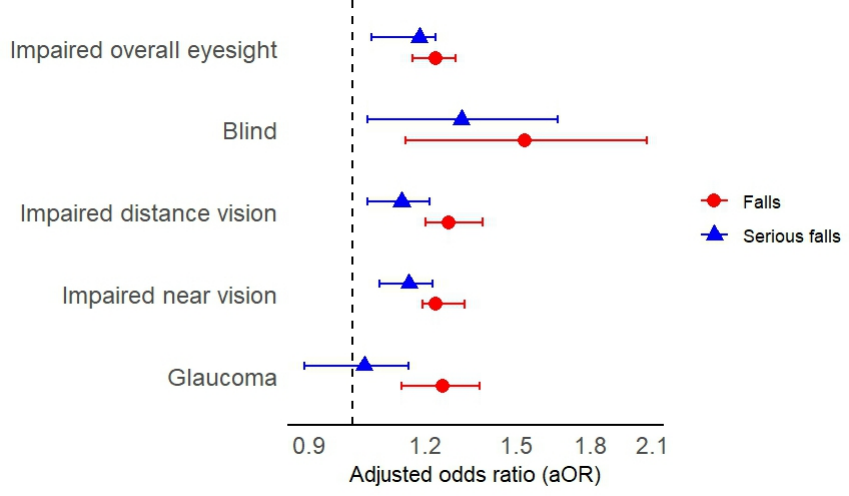
Forest plot illustrating the association between falls and ophthalmic parameters, based on a longitudinal multivariable model using Generalized Estimating Equation.

In the longitudinal analysis of the association between ophthalmic parameters with falls and serious falls, impaired overall eyesight, impaired distance vision, impaired near vision, and self-reported glaucoma all increased the odds of future falls with an OR of 1.23 (95% CI 1.16‐1.29; *P*<.001), 1.27 (95% CI 1.20‐1.38; *P*<.001), 1.23 (95% CI 1.19‐1.32; *P*<.001), and 1.25 (95% CI 1.13‐1.37; *P*<.001), respectively (see Table 6 and Figure 2). Impaired overall eyesight, distance vision, and near vision also increased the risk of serious falls in the subsequent 2 years with an OR of 1.18 (95% CI 1.05‐1.23; *P*<.001), 1.13 (95% CI 1.04‐1.21; *P*<.001), and 1.15 (95% CI 1.07‐1.22; *P*<.001), respectively. There was no association between self-reported glaucoma and the odds of future serious falls (*P*=.32).

**Table 6.** Longitudinal model showing the association between falls and ophthalmic parameters.

Type of falls	OR^a^ (95% CI)	*P* value	Adjusted OR (95% CI)^b^	*P* value
Falls				
Overall eyesight				
Not impaired	Ref^c^	Ref	Ref	Ref
Impaired	1.59 (1.53‐1.67)	<.001	1.23 (1.16‐1.29)	<.001
Blind	2.03 (1.65‐2.68)	<.001	1.53 (1.14‐2.07)	.005
Impaired distance vision	1.73 (1.63‐1.88)	<.001	1.27 (1.20‐1.38)	.001
Impaired near vision	1.69 (1.63‐1.75)	<.001	1.23 (1.19‐1.32)	<.001
Glaucoma	1.40 (1.23‐1.52)	<.001	1.25 (1.13‐1.37)	<.001
Serious falls				
Overall eyesight				
Not impaired	Ref	Ref	Ref	Ref
Impaired	1.17 (1.10‐1.23)	<.001	1.18 (1.05‐1.23)	<.001
Blind	1.45 (1.17‐1.93)	<.001	1.31 (1.04‐1.66)	.046
Impaired distance vision	1.20 (1.13‐1.26)	<.001	1.13 (1.04‐1.21)	.004
Impaired near vision	1.19 (1.13‐1.26)	<.001	1.15 (1.07‐1.22)	<.001
Glaucoma	1.15 (1.13‐1.17)	.039	1.03 (0.89‐1.15)	.32

a OR: odds ratio.

b Adjusted for age, sex, Hispanic ethnicity, race, education, marital status, hypertension, diabetes, heart disease, stroke, arthritis, smoking, alcohol, activity, psychiatric medication, vigorous activities, and household income.

c Reference.

### Analysis Between Mean Number of Falls and Eyesight

The mean number of falls was associated with the degree of self-reported vision impairment (see Table 7). The level of eyesight reported in the questionnaire was associated with the reported number of falls in the same survey cycle as well as the subsequent cycle (*P*<.001 for both). Approximately 2.57 (SD 4.58) falls per person who fell were observed in the group with excellent current eyesight compared to 3.70 (SD 5.33) for those with poor reported vision. Similarly, self-reported excellent overall eyesight had an average of 2.78 (SD 4.17) falls per person while poor overall eyesight had approximately 3.65 (SD 5.40) falls per person in the subsequent cycle.

**Table 7.** The association of the degree of visual impairment and mean number of falls.

Degree of impairment	Excellent	Very good	Good	Fair	Poor	*P* value
Cross-sectional, mean (SD)						
Overall eyesight	2.57 (4.58)	2.58 (3.84)	2.74 (4.05)	3.15 (4.59)	3.70 (5.33)	<.001
Distal eyesight	2.66 (4.16)	2.70 (4.04)	2.79 (4.05)	3.34 (4.94)	3.73 (5.26)	<.001
Near eyesight	2.59 (3.86)	2.65 (4.23)	2.77 (4.04)	3.2 (4.52)	3.88 (5.79)	<.001
Longitudinal, mean (SD)						
Overall eyesight	2.78 (4.17)	2.71 (4.10)	2.84 (4.18)	3.19 (4.72)	3.65 (5.40)	<.001
Distal eyesight	2.74 (4.13)	2.75 (3.95)	2.93 (4.40)	3.27 (4.89)	3.59 (5.08)	<.001
Near eyesight	2.66 (3.92)	2.72 (4.01)	2.91 (4.37)	3.28 (4.92)	3.62 (5.10)	<.001

## Discussion

### Principal Findings

In this large and diverse longitudinal population-based study of older adults, poor vision, including both distance and near vision, increased the odds of falling, and more severe vision loss was related to the number of falls. We additionally found that the presence of self-reported glaucoma was associated with falls.

### Comparison With Previous Studies

Many population-based studies have found associations between self-reported vision impairment and falls. The Survey of Health, Ageing, and Retirement in Europe (SHARE) study found that self-reported distance and near vision impairment were cross-sectionally and longitudinally associated with increased risk of falls [21]. The Beaver Dam Eye Study reported that people with worse binocular acuity had higher odds of 2 or more falls [12], while the EPIC-Norfolk eye study found significant associations between poor self-reported distance vision and falls independent of visual acuity [20]. Similarly, we demonstrate here that the presence of visual impairment, including overall eyesight, near vision, and distance vision, is all associated with significantly increased odds for falls and severe falls in both our cross-sectional and longitudinal analyses. Our results suggest that self-reported visual status is predictive of falls and serious falls.

Importantly, the degree of self-reported visual impairment was associated with the number of falls even after adjusting for potential confounders. When looking at the mean number of falls that occur per person for each level of reported vision, we found that though people with excellent vision had falls, the number increased significantly as vision worsened, with those with blindness having the highest risk of falls. This is true for both falls that occurred 2 years before and those that occurred in the following 2 years of the reported vision and does not take into account the fact that blindness likely reduces the number of steps taken.

This study also subclassified falls as serious based on whether a fall had any associated injuries requiring medical treatment. The incidence of serious falls increased with age and deterioration of eyesight, and we found the risk factors for falls and serious falls to be similar. Other studies, including a Finnish population-based study, have examined risk factors of major injurious falls and similarly found poor distant visual acuity to be a risk factor for major falls among disabled elder adults [22].

Visual input plays a critical role in coordinating and planning movement, as well as maintaining balance [23]. Individuals with poor vision are more likely to lose stability, alter their gait to avoid obstacles, and ultimately increase their risk of slips, trips, and falls [24]. Our study differs from previous research in this area, which has largely relied on data from previous falls and visual function assessments. By using self-reported data, our research offers a fresh perspective and highlights the potential of self-reported visual function data as a predictive tool for falls risk.

We also found that glaucoma is a risk factor for serious falls resulting in injury despite the fact that our definition of glaucoma was imperfect (based on self-report only). Any nondifferential misclassification in diagnosing glaucoma would have driven our associations toward the null, and this implies that the associations found were real and may have been underestimated. Previous studies have also reported glaucoma as an independent risk factor for falling [13]. Glaucoma severity has been reported as a risk factor for falls, with advanced glaucoma patients having the highest risk of falls compared to controls with no glaucoma and mild or moderate glaucoma [25]. While central vision and, therefore, visual acuity are often preserved in glaucoma until severe stages of disease, glaucoma patients experience changes in multiple other aspects of vision, including VF loss, decrease in contrast sensitivity, and loss of depth perception. All these visual measures have been shown to independently increase the risk of falling [13,14,26]. Indeed, the Salisbury Eye Evaluation found that loss in peripheral VF is a more important risk factor for falls than loss of central VF [14]. Patients with glaucoma may experience one or all visual deficits simultaneously, further increasing the risk of falling.

Studies have suggested various interventions to reduce the risk of falls, including mobility training, vision interventions to improve vision (eye examinations, cataract surgery, and prescription glasses) [27], risk factor assessment, and environmental modifications to make living environment less hazardous [28]. Visual impairment leads to physical impairment of mobility and balance control, increasing the risk of falls [29]. Glaucoma initially affects peripheral vision through retinal nerve fiber layer loss, which is associated with decreased postural stability; therefore, glaucoma patients may benefit from mobility training to reduce falling risks [30,31].

### Clinical Implications

Our results provide evidence to support the development of multifaceted fall prevention strategies. Specifically, our findings suggest that self-reported vision screening could be a valuable tool in identifying individuals at risk of falls. This screening could be easily integrated into telemedicine platforms or incorporated into standard clinical guidelines. Furthermore, we propose the development of a simple, score-based questionnaire that can be used to predict falls risk in older adults. Those with higher scores could be targeted for earlier intervention, thereby reducing their risk of falls.

### Limitations

There were several limitations to our study. All data used in this study were from self-reported questionnaires administered every 2 years and are susceptible to recall bias and recall error. Specifically, participants may have difficulty recalling their visual function or falls history, which could lead to inaccurate reporting. In addition, while self-reported visual function has been shown to correlate closely with objective visual function, it is also understood as a multidimensional measure that reflects some, but not all components of vision [19]. Since no eye examinations were done and objective visual function was not measured, we cannot determine which visual components contributed to participants’ self-reported visual function. This study is also limited by the specific questions collected as part of the HRS study. No questions were asked about the other leading causes of blindness and visual impairment in this older adult population, such as age-related macular degeneration and other neurological conditions that could affect falls, such as Parkinson disease, dementia, or epilepsy. Finally, we could not explore other known vision-related risk factors for falls such as contrast sensitivity, VF deterioration, depth perception, and stereoacuity [26]. Nonvision factors such as cognitive ability, balance, motor control, and degree of mobility, which are known to be associated with falls, were not evaluated since they were not included in the questionnaire. The omission of these factors may limit the generalizability of our findings and highlights the need for future studies to incorporate a more comprehensive set of measures.

### Conclusions

Our analysis of data from a large, longitudinal national survey of older adults demonstrated that vision-related risk factors, particularly the presence and degree of visual impairment and glaucoma, increase the rate of falls and serious falls. We additionally show that self-reported vision status correlates with the number of fall events both at the time of and occurring after the survey date. Our results support screening for the presence of visual impairment and signs of glaucoma in older adults to identify individuals at the highest risk of falling who might most benefit from fall prevention interventions.

## Supplementary material

10.2196/68771 Multimedia Appendix 1 Self-reported visual function and falls questions in the Health and Retirement Study (HRS).

## Abbreviations

aOR: adjusted odds ratio
HRS: Health and Retirement Study
OR: odds ratio
VF: visual field

## References

[1] (2021). Step safely: strategies for preventing and managing falls across the life-course. https://www.who.int/publications/i/item/978924002191-4.

[2] Bergen G, Stevens MR, Burns ER (2016). Falls and fall injuries among adults aged ≥65 years - United States, 2014. MMWR Morb Mortal Wkly Rep.

[3] Moreland B, Kakara R, Henry A (2020). Trends in nonfatal falls and fall-related injuries among adults aged ≥65 years - United States, 2012-2018. MMWR Morb Mortal Wkly Rep.

[4] Rubenstein LZ, Josephson KR (2002). The epidemiology of falls and syncope. Clin Geriatr Med.

[5] Tinetti ME (2003). Clinical practice. Preventing falls in elderly persons. N Engl J Med.

[6] (2023). Facts about falls. https://www.cdc.gov/falls/data-research/facts-stats/index.html.

[7] Wang H, Naghavi M, Allen C (2016). Global, regional, and national life expectancy, all-cause mortality, and cause-specific mortality for 249 causes of death, 1980–2015: a systematic analysis for the Global Burden of Disease Study 2015. The Lancet.

[8] Close J, Ellis M, Hooper R, Glucksman E, Jackson S, Swift C (1999). Prevention of falls in the elderly trial (PROFET): a randomised controlled trial. Lancet.

[9] Ambrose AF, Paul G, Hausdorff JM (2013). Risk factors for falls among older adults: a review of the literature. Maturitas.

[10] Dhargave P, Sendhilkumar R (2016). Prevalence of risk factors for falls among elderly people living in long-term care homes. Journal of Clinical Gerontology and Geriatrics.

[11] Patil S, Suryanarayana S, Shivraj N, Murthy N, Dinesh R (2015). Risk factors for falls among elderly: a community-based study. Int J Health Allied Sci.

[12] Klein BEK, Moss SE, Klein R, Lee KE, Cruickshanks KJ (2003). Associations of visual function with physical outcomes and limitations 5 years later in an older population: the Beaver Dam eye study. Ophthalmology.

[13] Ivers RQ, Cumming RG, Mitchell P, Attebo K (1998). Visual impairment and falls in older adults: the Blue Mountains Eye Study. J Am Geriatr Soc.

[14] Freeman EE, Muñoz B, Rubin G, West SK (2007). Visual field loss increases the risk of falls in older adults: the Salisbury eye evaluation. Invest Ophthalmol Vis Sci.

[15] Nevitt MC, Cummings SR, Kidd S, Black D (1989). Risk factors for recurrent nonsyncopal falls. A prospective study. JAMA.

[16] Campbell AJ, Borrie MJ, Spears GF (1989). Risk factors for falls in a community-based prospective study of people 70 years and older. J Gerontol.

[17] Lord SR, Ward JA, Williams P, Anstey KJ (1994). Physiological factors associated with falls in older community‐dwelling women. J American Geriatrics Society.

[18] Fisher GG, Ryan LH (2018). Overview of the health and retirement study and introduction to the special issue. Work Aging Retire.

[19] El-Gasim M, Munoz B, West SK, Scott AW (2013). Associations between self-rated vision score, vision tests, and self-reported visual function in the Salisbury Eye Evaluation Study. Invest Ophthalmol Vis Sci.

[20] Yip JLY, Khawaja AP, Broadway D (2014). Visual acuity, self-reported vision and falls in the EPIC-Norfolk Eye study. Br J Ophthalmol.

[21] Ogliari G, Ryg J, Qureshi N, Andersen-Ranberg K, Scheel-Hincke LL, Masud T (2021). Subjective vision and hearing impairment and falls among community-dwelling adults: a prospective study in the Survey of Health, Ageing and Retirement in Europe (SHARE). Eur Geriatr Med.

[22] Koski K, Luukinen H, Laippala P, Kivelä SL (1998). Risk factors for major injurious falls among the home-dwelling elderly by functional abilities. A prospective population-based study. Gerontology.

[23] Manchester D, Woollacott M, Zederbauer-Hylton N, Marin O (1989). Visual, vestibular and somatosensory contributions to balance control in the older adult. J Gerontol.

[24] Buckley JG, Panesar GK, MacLellan MJ, Pacey IE, Barrett BT (2010). Changes to control of adaptive gait in individuals with long-standing reduced stereoacuity. Invest Ophthalmol Vis Sci.

[25] Bhorade AM, Perlmutter MS, Sabapathypillai SL, Goel M, Wilson B, Gordon MO (2021). Rate of falls, fear of falling, and avoidance of activities at-risk for falls in older adults with glaucoma. Am J Ophthalmol.

[26] Mehta J, Czanner G, Harding S, Newsham D, Robinson J (2022). Visual risk factors for falls in older adults: a case-control study. BMC Geriatr.

[27] Zhang XY, Shuai J, Li LP (2015). Vision and relevant risk factor interventions for preventing falls among older people: a network meta-analysis. Sci Rep.

[28] Sotimehin AE, Yonge AV, Mihailovic A (2018). Locations, circumstances, and outcomes of falls in patients with glaucoma. Am J Ophthalmol.

[29] Zwierko T, Jedziniak W, Florkiewicz B (2021). The consequences of glaucoma on mobility and balance control in the older adults: a cross-sectional study. J Aging Phys Act.

[30] O’Connell C, Mahboobin A, Drexler S (2017). Effects of acute peripheral/central visual field loss on standing balance. Exp Brain Res.

[31] Black AA, Wood JM, Lovie-Kitchin JE, Newman BM (2008). Visual impairment and postural sway among older adults with glaucoma. Optom Vis Sci.

