# Supplementary material for: The Impact of Vision Impairment on Self-Reported Falls Among Older US Adults: Cross-Sectional and Longitudinal Study
Source: JMIR Aging. 2025 Jul 31;8:e68771. doi: 10.2196/68771 (PMC12313157; doi:10.2196/68771)
Supplement: Multimedia Appendix 1 [file aging-v8-e68771-s001.docx]

**Table S1.** Self-reported visual function and falls questions in HRS.

| **Variables** | **Question** | **Response** |
| --- | --- | --- |
| Overall eyesight | Is your eyesight excellent, very good, good, fair, or poor (using glasses or corrective lenses as usual) | Excellent, very good, good, fair, poor, or legally blind |
| Distance vision | How good is your eyesight for seeing things at a distance, like recognizing a friend across the street (using glasses or corrective lenses as usual)? | Excellent, very good, good, fair, or poor |
| Near vision | How good is your eyesight for seeing Excellent, very good, good, fair, or poor Study participant things up close, like reading ordinary newspaper print (using glasses or corrective lenses as usual)? | Excellent, very good, good, fair, or poor |
| Glaucoma | Has a doctor ever treated you for glaucoma? | Yes or No |
| Self-reported Falls | Have you fallen down in the last two years? | Yes or No |
| Self-reported serious falls | In that fall, did you injure yourself seriously enough/In any of these falls, did you injure yourself seriously enough to need medical treatment? | Yes or No |
